# Treatment of tissue necrosis with hyperbaric oxygen therapy in a patient with pseudomonas endophthalmitis and orbital cellulitis: a case report

**DOI:** 10.22336/rjo.2023.33

**Published:** 2023

**Authors:** Ismail Uyanik, Husna Topcu, Kubra Serefoglu Cabuk, Ayse Cetin Efe, Mehmet Goksel Ulas

**Affiliations:** * University of Health Sciences, Istanbul, Turkey; Beyoglu Eye Training and Research Hospital, Istanbul, Turkey

**Keywords:** endophthalmitis, evisceration, hyperbaric oxygen therapy, orbital cellulitis, Pseudomonas aeruginosa

## Abstract

**Purpose:** This report presents the treatment of tissue necrosis after evisceration with hyperbaric oxygen therapy (HBOT) in a patient with pseudomonas endophthalmitis and orbital cellulitis.

**Methods:** A 49-year-old woman was admitted to our clinic with severe pain and vision loss after cataract surgery 3 days before, and pars plana vitrectomy 2 days before for endophthalmitis, in another hospital. Examination findings included limbal perforation, orbital cellulitis findings, and loss of light perception in the right eye. The patient, who received evisceration surgery and antibiotic treatment, showed loosening of the conjunctival sutures and necrosis in the conjunctiva, tenon, and sclera on the 9th postoperative day. The necrotic tissues were surgically debrided and the patient was referred to HBOT.

**Results:** With HBOT and antibiotherapy, signs of inflammation regressed, healing on the conjunctival surface was accelerated, and prosthesis was suitable for use.

**Conclusions:** Hyperbaric oxygen therapy is a treatment method that plays an active role in the healing of necrotic tissues by increasing the oxygenation and vascularization of the tissue.

## Introduction

*Pseudomonas aeruginosa* is a gram-negative rod commonly known as an agent of nosocomial infections. The most common ocular infection of *P. aeruginosa* is contact lens wear-induced keratitis [**1**]. *P. aeruginosa* endophthalmitis often results in a poor visual prognosis despite intravitreal and systemic antibiotic therapy and may result in enucleation or evisceration in 64% [**2**]. Despite endophthalmitis treatment, catastrophic soft tissue infections such as orbital cellulitis, ocular necrotizing fasciitis, periorbital gangrene, and eyelid necrosis caused by *P. aureginosa* have been reported [**3**,**4**]. 

Hyperbaric oxygen therapy (HBOT) can be primary or adjunctive therapy for many disorders, including central retinal artery occlusion, periocular necrotizing fasciitis, ocular and periocular gas gangrene, and some eye diseases, such as periorbital reconstructive surgery [**5**]. HBOT can enhance wound healing by increasing tissue oxygenation, improving phagocytosis, and increasing leukocyte microbial killing ability [**6**]. 

We aimed to present a patient who underwent evisceration surgery for endophthalmitis and orbital cellulitis after cataract surgery caused by *P. aeruginosa*.

## Case report

**Fig. 1:**
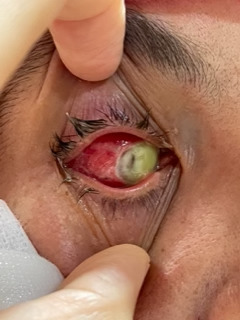
Image of the patient at presentation with limbal perforation

A forty-nine-year-old female patient was admitted to our emergency department with complaints of pain in the right eye and loss of vision. She had cataract surgery in her right eye three days before at another hospital. One day before, pars plana vitrectomy was performed due to endophthalmitis in the same hospital. On examination, edema and hyperemia of the upper and lower eyelids were present. Her visual acuity was no light perception in the right eye and 20/ 20 in the left eye. The conjunctiva was hyperemic, the cornea was completely infiltrated, and there was a limbal perforation at the 10 o’clock position (Seidel sign +) (**Fig. 1**). The details of the anterior chamber could not be distinguished in the slit-lamp examination of the OD. The left eye was completely normal.

The culture result of the endophthalmitis agent could not be obtained. Due to excruciating pain and loss of light perception, emergency evisceration was performed on the right eye with four scleral petals. During the operation, four relaxing posterior sclerotomies, and peripapillary detachment of the optic nerve were performed with the implantation of a 21 mm acrylic sphere. No perioperative complications developed. The removed intraocular materials were sent for microbiological and pathological evaluation. Microbiological culture results showed *P. aeruginosa* sensitive to meropenem, tobramycin, and amikacin, and intermediate to levofloxacin and ceftazidime. The patient received parenteral treatment with meropenem (3 x 1000 mg), levofloxacin (1 x 750 mg), vancomycin (2 x 1000 mg), and topical ceftazidime (50 mg/ ml, 6 x 1) for ten days.

**Fig. 2:**
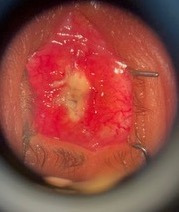
Conjunctival and scleral necrosis appearance

Periocular inflammation decreased with the effect of systemic and topical antibiotics in the postoperative period. However, on the 9th postoperative day, loosening of the conjunctival sutures, avascular conjunctiva, and tenon were observed at the wound edges (**Fig. 2**). The patient was re-operated for debridement of necrotic tissues. Necrotic tissues on the anterior margins of scleral petals were debrided. The vital sclera, tenon, and conjunctiva were sutured in layers with 6/ 0 vicryl sutures to cover the acrylic sphere.

To prevent postoperative necrosis and implant extrusion, the patient was referred to HBOT (2-3 absolute pressure, 30 min./ 10 sessions). He received intravenous levofloxacin treatment (1 x 750 mg) for ten days again. On the 10th day of treatment, a healing process was observed with intact conjunctival sutures and a well-vascularized pinkish socket, and neither inflammation nor necrosis was detected.

**Fig. 3:**
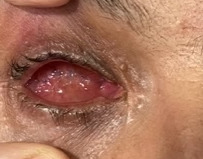
Intact conjunctival sutures and a well-vascularized pinkish socket

**Fig. 4:**
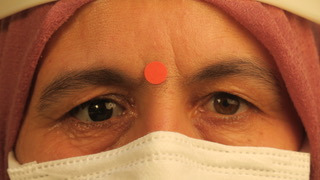
Patient’s view with right prosthetic eye

At our last visit, the socket improved due to combined HBOT and systemic antibiotic therapy (**Fig. 3**). An appropriate prosthesis was applied to the patient two months after surgery (**Fig. 4**).

## Discussion

*Pseudomonas aeruginosa* infection is caused by contaminated ophthalmic solutions, such as intraocular lens solutions, trypan blue, and the contamination of phaco probes in an ophthalmic setting [**7**]. *P. aeruginosa* produces elastases and exotoxins so that the cornea, anterior chamber, and vitreous cavity may be damaged rapidly and permanently [**8**]. After this infection, uncontrolled inflammation and globe disorganization can lead to loss of the eye. The enucleation/ evisceration surgery rate after the pseudomonas endophthalmitis that is induced by cataract surgery is 64% [**2**]. In the presence of findings such as edema, erythema, and inflammation of the eyelids, proptosis, chemosis, increased intraocular pressure, and decreased ocular motility, together with endophthalmitis, orbital cellulitis clinic should be considered.

Evisceration offers many advantages over enucleation in patients with endophthalmitis, such as better postoperative fornix and implant motility, easier prosthetic placement, and overall better cosmetic outcomes. Sphere implantation during evisceration surgery is a matter of debate in cases of endophthalmitis. Some surgeons prefer complete regression of inflammation to prevent implant loss. Some studies have reported that globe implantation in the same session can reduce hospital stay [**9**]. We also performed sphere implantation in the first surgery.

HBOT is commonly used for the treatment of damaged tissue oxygenation. The use of HBOT has also been reported in ocular conditions involving ischemia, including retinal artery and vein occlusions, anterior segment ischemia, and scleral melt [**10**]. In scleral ischemia, HBOT is proposed to stimulate scleral angiogenesis and fibroblast proliferation. Pseudomonas’s exotoxins can cause damage to the vascular supply of ophthalmic tissue. The main part of the treatment of infection is to transport the oxygen and antibiotic to the tissue and increase the recovery rate. Our patient did not have any known immunocompetent disease. There was aggressive endophthalmitis and corneal perforation due to possible contamination in cataract surgery. On the 9th day after emergency evisceration, conjunctival sutures were opened and scleral necrosis was present. Despite this rapid and catastrophic course, we assumed that HBOT combined with antibiotic therapy and surgical debridement affects wound healing. Recovery of avascular and damaged tissue was accelerated after HBOT and vascularization of the tissue was increased.

## Conclusion

To our best knowledge, this is the first report of successful results of using HBOT as adjunctive therapy for scleral necrosis following evisceration surgery secondary to pseudomonas endophthalmitis. What should be emphasized is the importance of HBOT support in the recovery of tissue necrosis that develops in the postoperative period. We believe that it is an effective and safe treatment that can be used in similar cases. 

**Conflict of Interest statement**

The author(s) declare no potential conflicts of interest with respect to the research, authorship, and/ or publication of this article.

**Informed Consent and Human and Animal Rights statement**

Informed consent was obtained from the subject.

**Authorization for the use of human subjects**

Ethical approval: The research related to human use complies with all the relevant national regulations, institutional policies, is in accordance with the tenets of the Helsinki Declaration. Ethical approval was not required. A written, informed consent was obtained from the patient for the publication of this case report and any accompanying images.

**Acknowledgements**

None.

**Sources of Funding**

The author(s) received no financial support for the research, authorship, and/ or publication of this article.

**Disclosures**

None.
